# Food Patterns According to Sociodemographics, Physical Activity, Sleeping and Obesity in Portuguese Children

**DOI:** 10.3390/ijerph7031121

**Published:** 2010-03-17

**Authors:** Pedro Moreira, Susana Santos, Patrícia Padrão, Tânia Cordeiro, Mariana Bessa, Hugo Valente, Renata Barros, Vitor Teixeira, Vanessa Mitchell, Carla Lopes, André Moreira

**Affiliations:** 1 Faculty of Nutrition and Food Sciences, University of Porto, Rua Roberto Frias, 4200-465 Porto, Portugal; E-Mails: patriciapadrao@fcna.up.pt (P.P.); taniajcordeiro@gmail.com (T.C.); bessa.mariana@gmail.com (M.B.); renatabarros@fcna.up.pt (R.B.); vhugoteixeira@fcna.up.pt (V.T.); 2 Research Centre on Physical Activity and Health, University of Porto, Rua Dr. Plácido Costa, 91, 4200-450 Porto, Portugal; E-Mail: hugovalente@fcna.up.pt; 3 Institute of Public Health, University of Porto, Rua Prof. Hernâni Monteiro 4200-319 Porto, Portugal; E-Mail: carlal@med.up.pt; 4 Department of Hygiene and Epidemiology, Faculty of Medicine, University of Porto Medical School, Rua Prof. Hernâni Monteiro 4200-319 Porto, Portugal; E-Mail: sumoreirasantos@gmail.com; 5 Department of Psychology, Glasgow Caledonian University, Cowcaddens Road, Glasgow, Scotland, UK; E-Mail: vanessa.mitchell@gcal.ac.uk; 6 Department of Immunology, Faculty of Medicine, University of Porto, Rua Prof. Hernâni Monteiro 4200-319 Porto, Portugal; E-Mail: andremoreira@med.up.pt; 7 Department of Immunoallergology, Hospital of São João, Rua Prof. Hernâni Monteiro 4200-319 Porto, Portugal

**Keywords:** food patterns, children, obesity, education, physical activity, sleeping

## Abstract

Our study aimed to describe the association between food patterns and gender, parental education, physical activity, sleeping and obesity in 1976 children aged 5−10 years old. Dietary intake was measured by a semi quantitative food frequency questionnaire; body mass index was calculated and categorized according to the IOTF classification. Factor analysis and generalized linear models were applied to identify food patterns and their associations. TV viewing and male gender were significant positive predictors for fast-food, sugar sweetened beverages and pastry pattern, while a higher level of maternal education and longer sleeping duration were positively associated with a dietary patterns that included fruit and vegetables.

## 1. Introduction

Some studies have described the linkages between health or disease and children’s diet, in terms of food groups (e.g., fruit and vegetables [1]) or the content of single nutrients (e.g., calcium [2] or fibre [3]), or single foods [4]. However, given the complexity of the relationships between intakes of various nutrients, bioactive components or foods, results describing the effects of consumption of single nutrients or foods on a given health outcome may be misleading. To overcome these limitations, food pattern analysis is studied as it considers the combinations of foods. One major approach to identify food patterns of a population from which dietary data are collected is the use of specific statistical methods, such as factorial analysis [5].

The patterns of food consumption are shaped by demographic, economic, and cultural determinants, varying also with other lifestyle characteristics, such as physical activity or TV viewing. Although some studies have aggregated foods and lifestyle behaviors in different patterns [6,7], evidence about the associations between dietary patterns and sociodemographic factors, physical activity, sleeping, and obesity in children is scarce [6–10]. The recognition of food patterns in the population and their possible relations with lifestyle variables could provide a useful basis for developing, monitoring and targeting food and nutrition policy for school children.

Therefore, the present study aimed to describe food patterns, according to gender, parental education, physical activity, and obesity, in Portuguese children.

## 2. Material and Methods

### 2.1. Study Design and Participants

This was a cross-sectional study, approved by each School Committee and by the Ethical committee of São João Hospital, Porto, carried out between October 2006 and March 2007.

The data were derived from a community-based survey of children. Forty from the 59 elementary schools from the city of Porto, Portugal were randomly selected, of which 35 accepted to participate. Within those schools all children were invited to participate (n = 5,736). Letters were distributed to all parents outlining the aims of the study along with a consent form. Fifty-eight percent of parents signed and returned the filled-out form, therefore the sample consisted of 3,327 children. Anthropometric measurements were taken from all consenting children, and questionnaires surveying sociodemographic characteristics, physical activity and dietary intake, were distributed to parents, of which 2,462 were returned (43% of the total subjects). Children excluded from the final analysis included three subjects that presented congenital malformations, another 242 due to incorrect questionnaire, and 241 children for presenting extreme values of energy intake considering the Willet’s cut-off [11], leaving a final sample of 1,976 children from 5−10 years old, of which 991 (50.2%) were girls.

### 2.2. Social, Demographic and Family Characteristics’

The questionnaire contained information about the gender, the age of children, and the education of the parents, recorded in five categories of years: 0, 1−4, 5−9, 10−12, and more than 12 years of formal education. This information was further grouped for analysis into three categories: up to 9 years, 10−12 years, and more than 12 years of education.

### 2.3. Physical Activity

In order to assess the level of physical activity of children, the questionnaire included: the time spent watching TV/video during most days of the week (recorded in five categories: less than 1 h/day, 1−2 h/day, 2−4 h/day, 4−6 h/day, and more than 6 h/day; and subsequently grouped for the analysis into two categories: less than 2 h/day; and 2 h/day or more); sleeping duration (recorded in hours/minutes per day, and classified for the analysis into three categories: less than 8 h/day; 9 h/day; and 10 h/day or more); and practice of sports activities besides the physical education classes at school (recorded in six frequency categories: “never”, “at least once per month”, “between once per month and once per week”, “2−3 times per week”, “4−6 times per week”, or “everyday”; and subsequently grouped for the analysis into three categories: less than 2 times/week; 2−3 times/week; 4 times/week or more).

### 2.4. Dietary Intake

Parents were asked to report the frequency of their child’s food consumption by completing a self-administered, semi-quantitative food frequency questionnaire (FFQ), validated for Portuguese adults [12]. The FFQ is an 86-item questionnaire that assesses usual dietary intake over the previous 12 months, including food groups and beverages. Food intake was calculated by weighting one of the nine possibilities of frequency of consumption (from “never or less than once per month”, to “six or more times a day”), by the weight of the standard portion size of the food-item. Energy and nutritional intake were estimated using an adapted Portuguese version of the nutritional analysis software Food Processor Plus (ESHA Research Inc., Salem, OR, USA).

### 2.5. Anthropometric Measurements

In each school, two trained nutritionists performed anthropometric measurements according to standardized procedures [13]. Height was measured using a stadiometer, with the head in the Frankfort plane, and weight was measured using an electronic scale with an error of ±100 g (Seca, Model 703, Germany), without shoes and using light indoor clothing. BMI was computed and the prevalence of underweight, normalweight, overweight and obesity were calculated according to the International Obesity Task Force (IOTF), making a correspondence between the traditional adult cut-offs and specific values for children considering sex and age [14]. Underweight (2.5% and 2.8% of girls and boys, respectively) and normal weight children were grouped into one category (reference) for the generalized linear model analysis.

### 2.6. Statistical Analysis

Mean and standard deviations (SD) were used to describe continuous variables (except for physical activity, and for dietary intake data that were presented as median and interquartile range given the non-normal distribution). Student’s t-tests, Mann-Whitney U, and Chi-square tests were used to compare several variables between genders; a 0.05 level of significance was considered.

To identify dietary patterns within the study population, multivariate statistical techniques are applicable, with factor analysis being one technique that may be used [15]. Factor analysis characterizes the covariance structure among variables in terms of a few underlying factors. In our study, factor analysis was performed to identify the foods kept under the factorial solution. We used a principal component analysis to estimate the number of factors emerging, and assessed the appropriateness of the correlation matrices for factor analysis. Kaiser’s measure of sampling adequacy values were above 0.6, which is required for a good factor analysis [16]. To identify the number of factors to be retained, we employed the eigenvalue higher than 1.0, which is widely used in factor analysis and is based on the rationale that each factor retained should explain more variance than a single original variable in the data set. After a Varimax rotation of the factors, food items with absolute factor loadings above 0.30 were considered as significantly contributing to a cluster. Factor loadings in the factorial analysis can be interpreted as correlation coefficients between food items.

The first factorial analysis considered the 86 items in the FFQ, but the solution presented 30 factors, and the rotation failed to converge, which made it difficult to interpret. We removed from the 86-items the alcoholic beverages (these beverages were not consumed by more than 99%), and the remaining items were further categorized into 29 food groups, according to nutritional composition similarities: vegetables, vegetable soup, fresh fruit, canned fruit, olives, nuts, fish (including seafood or shellfish), meat (beef, lamb, pork, offal, and poultry), processed meats (e.g., sausages, ham, smoked ham, chorizo), eggs, olive oil, vegetable oils, butter, margarine, milk, yogurt, cheese, milk-based pudding (e.g., custard, vanilla), ice cream, starches (pasta/spaghetti, potato/chips, and rice), bread, ready-to-eat cereals, sugar sweetened beverages, coffee, tea, fast food (pizza, hamburgers, and snack fried foods), pastry (cake, sugar biscuits/wafers, muffins, chocolate candy, and sugar), crackers/cookies (approximately 20% of sugar or less), and pulses.

A subsequent factorial analysis was employed considering the 29 food groups, and five of them (canned fruit, olives, nuts, tea, and coffee) did not load on any retained factor. These last five food groups were excluded in the final factorial analysis.

As in other studies [7,15], pattern scores were then calculated considering the sum of all ingested dietary items quantities (g/day) multiplied by their correspondent factor loadings. These clusters scores of food patterns were then used as dependent variables in generalized linear models considering children nested within schools, age, gender, normalweight, overweight, obesity, TV viewing, sleeping, frequency of sports activities, parents education, and energy intake as independent variables. Variables with a *p* value < 0.05 are presented in the linear model for each food pattern.

The data analysis was performed using SPSS ®, Version 17.0 (SPSS Inc; Chicago, IL).

## 3. Results

### 3.1. Participants’ Characteristics

Subjects included 991 girls and 985 boys with a mean age of 7.5 (SD = 1.2) years for both genders (p = 0.319). Prevalence of overweight/obesity was 38.8% in girls and 38.6% in boys (p = 0.488). Energy and nutritional intake by gender are presented in Table 1. For both genders, the diet was high in fat, particularly saturated fat, sugars and protein, and low in total carbohydrates and dietary fiber. Boys exhibited significantly higher consumption of dietary fiber (25.7 *versus* 24.1 g/day, p = 0.001) and total carbohydrates (51.7% *versus* 51.0% of total energy, p = 0.017), and lower consumption of protein (18.1% *versus* 18.5% of total energy, p = 0.025). Median and interquartile range intakes of the different food groups is shown in Table 1.

Girls, compared to boys, consumed significantly lower amounts of meat (91 *versus* 96 g/day), bread (47 *versus* 52 g/day), and SSB (106 *versus* 132 g/day), and higher amounts of vegetables (103 *versus* 91 g/day, p = 0.024, Table 1); girls were also less frequently involved in physical activities than boys (58.8% of girls reported exercise < 2 times/week *versus* 46.8% of boys, p < 0.001). The median of sleep duration was 9 h/day for both genders (Table 2). The proportions of children whose parents reported to spent two or more hours watching TV/video during most days of the week were 10.3%, of boys, and 11.1% of girls. Almost half of the parents reported to have attained more than 12 years of education (Table 2).

### 3.2. Associations between Food Patterns and Sociodemographic Characteristics, Physical Activity, Sleeping Pattern and Weight

The factorial analysis exhibited eight patterns (factors) that satisfactorily described the distributions of ingested foods groups in children. The eight patterns (Table 3) explained 48% of the variance, and positive loadings in each pattern were as follows: pattern 1 (9.9% of variance) exhibited high positive loadings on foods of vegetable origin (vegetables, pulses, and fruit), and olive oil; pattern 2 (7.1% of variance), fish, meat, processed meats, eggs, and starchy foods; pattern 3 (5.9% of variance), vegetable soup, olive oil, butter, starchy foods, and bread; pattern 4 (5.8% of variance), fast-food, SSB, and pastry; pattern 5 (5.3% of variance), olive oil, butter, and margarine; pattern 6 (5.0% of variance) was mostly due to dairy foods (yoghurt, cheese, and ice cream); pattern 7 (4.7% of variance), pastry and crackers/cookies; pattern 8 (4.3% of variance), was related to the combination of milk, milk pudding (chocolate, vanilla), and ready-to-eat cereals intake.

In generalized linear models, children were nested within schools, and independent variables included children’s gender (omitted/reference category: female), overweight, obesity (omitted/reference category: under/normal weight), TV viewing (omitted/reference category: up to 2 h/day), sleeping (omitted/reference category: ≤8 h/day), frequency of sports activities (omitted/reference category: <2 times/week), and maternal and paternal education (omitted/reference category: up to 9 years), adjusting for children’s age and energy intake.

Regression parameters for each cluster are presented in Table 4. Scores were predicted in: pattern 1, positively, by sleeping for ≥10 h/day and maternal education >12 years; pattern 2, positively, by male gender, TV viewing for ≥2 h/day, sleeping for ≥10 h/day, sports activities for ≥2 times/week, and maternal education >12 years; pattern 3, positively, by male gender, sleeping for ≥9 h/day, sports activities for ≥ 2 times/week, and maternal education >12 years, and negatively, by TV viewing for ≥2 h/day; pattern 4, positively, by male gender, and TV viewing for ≥2 h/day, and negatively, by sleeping for ≥9 h/day, and maternal education >12 years; pattern 5, positively, by TV viewing for ≥2 h/day; pattern 6, positively, by obesity, and sports activities for ≥4 times/week; pattern 7, positively, by male gender, and negatively, by obesity, and sports activities for ≥4 times/week; and pattern 8, positively, by sleeping for 9 h/day.

## 4. Discussion

This study showed that sleep duration, maternal education and the practice of sports activities were positively associated with dietary patterns mainly characterized by foods of plant origin (patterns 1 and 3), while TV viewing, lower maternal education and lower sleep duration, were positively associated with a dietary pattern that included foods rich in fat and added sugar (pattern 4), after adjustment for confounders (age and energy intake).

Eight distinct patterns were identified in the factor analysis. Pattern 1 included micronutrient-rich and low energy-dense foods—vegetables, fruits, and pulses—and olive oil, and emerged as the strongest food pattern among children living in a country where the Mediterranean diet still exhibits protective health effects in adults [17]. However, as Lioret *et al*. [7] argue, comparison of food patterns between studies is not simple, due to differences in the methodological approach to dietary assessment, the redistribution of foods into categories or food groups, the food pattern analysis, the number of groups identified for entry into the analysis, the methods of rotation of axes, if any, the number of patterns retained for analyses, and the statistical analysis techniques used. Nevertheless, some similarities can be observed between our results and the findings of Lioret *et al*. [7] such as the positive relationships between maternal education and food patterns 1 (vegetables, fruit, pulses, and olive oil) and 3 (vegetable soup, olive oil, starchy foods, bread, and butter), and with the findings from Aranceta *et al*. [8] and Northstone [10], in which the healthy patterns (that included higher intakes of vegetables and fruits), were positively associated with the level of education of the mother.

Maternal educational attainment of more than 12 years was a significant predictor of four food patterns, emphasizing the importance that education may have in choosing foods of plant origin (food patterns 1 and 3), and avoiding foods that may be energy dense and micronutrient poor (e.g., pastry, in food pattern 4). The role of the mothers is of particular interest on children’s eating behavior, because they generally spend significantly more time than fathers in direct interactions with their children [18]. Confidence in a significant positive causal link between education and food habits reinforces that public policy should stress education as a mean for improving healthy behaviors. Education may influence food choice by facilitating or constraining one’s ability to understand the information communicated in nutrition education or on food labels [19,20], and to facilitate access to an affordable supply of fresh, nutrient-rich foods [21], such as those observed in patterns 1 and 3.

The food patterns 2 (animal and starchy foods), 3 (vegetable soup, olive oil, butter, bread, and starchy foods), and 4 (fast-food, SSB, and pastry) were positively predicted by male gender. Previous research has documented that boys exhibit higher consumption than girls of processed meats, white bread, chips, and pasta, and less healthy food choices than girls, showing higher preferences for burgers, pizza [10], and other fatty and sugary foods [22]. TV viewing for more time than recommended by the American Academy of Pediatrics (up to 2 h/day) [23] was also a positive predictor of fast-food, SSB and pastry consumption (food pattern 4), and other energy-dense foods such as fats/oils (food pattern 5). Increased TV viewing time, a particular form of very sedentary behavior, has been consistently associated with unhealthful eating choices [24,25] including higher consumption of pizza, snack foods and sodas [26]. We identified some resemblance between our results and those reported in Spanish children aged 3–14 years [8] by the association between TV viewing for more than 2 h/day and low maternal education, with snack foods (e.g., soft drinks) intake.

Although more research needs to be conducted specifically in regard to the effects of fast food on health, public health recommendations presently include the need to limit fast food consumption [27], and to decrease added sugars in beverages and foods [28].

The consumption of sugar-sweetened beverages as a key contributor to the epidemic of overweight and obesity in children and adolescents has been strongly debated [29,30]. However, the current evidence fails to demonstrate that SSB consumption have a large and convincing effect on the BMI of children or adolescents [31], and the present study also failed to show any significant association between pattern 4 and overweight or obesity.

In this study, the prevalence of overweight and obesity was very high, emphasizing the epidemic state of overweight/obesity in Portuguese children and adolescents [32]. Despite considerable research effort on the nutritional etiology of childhood obesity, the roles of foods remain controversial, and food pattern studies have generally revealed few if any consistent associations between specific food selection patterns and obesity [7,33]. Morgan *et al*. [34] analyzed intakes of sugar and snacks in 5 to 18 years old children, and found no consistent associations between weight status and food patterns. In addition, there is no convincing evidence that the diet of obese people is rich in sweet foods [35]. Furthermore, in the present study, obesity was negatively associated with pastry, and crackers/cookies (food pattern 7). The latter results may reflect under-reporting or avoidance of foods recognized as high energy-dense such as those from food pattern 7, in obese children. However, the interpretation of these results is difficult because they relied on self-report data, and the evidence that particular foods may be associated with obesity is problematic because obese children may select or avoid certain foods as a result of their present weight status.

Obese children also exhibited a positive association with yoghurt, cheese, and ice cream intake (food pattern 6), which may be consumed as part of family snacks or desserts. In a very elegant study, Coates *et al.* [36] collected anthropometric data on family members and categorized stored food at home, showing that the degree of overweight in fathers was positively correlated with the number of dessert items. If we consider that obesity tend to aggregate within families [37], and children’s eating behavior is influenced by family lifestyle, values, and beliefs, this may provide indirect support for the observed higher consumption of foods (yoghurt, cheese, and ice cream) that typically may be eaten by Portuguese children as savory desserts or snacks.

Larger eaters due to higher physical activity may eat more of everything [33], but the present study showed a negative association between frequency of sports activities and pastry/cookies consumption (food pattern 7), and a positive association with a meat/fish/eggs/starchy foods (food pattern 2), vegetable soup/olive oil/butter/starchy foods (food pattern 3), and yoghurt/cheese/ice cream (food pattern 6). Although these results are not strictly comparable to other findings, positive associations between physical activity and food choices such as fruits and vegetables [38,39], and cheese, yoghurt, meat, processed meat, fish, and starchy foods consumption [10] have been reported in children with increased levels of physical activity.

Food pattern 2 aggregated fish, meat, processed meat, eggs, and starchy foods consumption, which may also contribute to an adequate nutrition in childhood [40]. Taking into account the Portuguese Food Wheel recommendations for the total amount of meat, fish and eggs (ranging from 45 to 135 g) [41], the median daily servings of those foods reported in this study was relatively high. Furthermore, the interquartiles ranges for fish, meat, and eggs intake were relatively large, suggesting that the sample included both high and low consumers of these foods. The nutritional meaning of this behavior is difficult to establish since processed meats and these animal foods should be consumed in balanced amounts, and current dietary guidelines include the limitation of animal foods intake, such as red meat [42]. Longer sleep duration was positively associated with the latter food pattern, and food patterns that included fruit, vegetables and olive oil (pattern 1), vegetable soup, starchy foods, and olive oil (pattern 3), and milk, milk-based pudding and ready to eat cereals (food pattern 8). By showing these positive associations, longer sleep duration emerged as a lifestyle characteristic related to these nutrient-rich foods and to healthy eating habits [43,44]. Sleep duration was also negatively associated with fast-food, SSB, and pastry (pattern 4). The inverse association between sleep length and the consumption of energy-rich foods have also been found in children from Finland [45].These findings are important because sleep curtailment has become an endemic behavior in modern society [46] and about 50% of children may sleep less than 9 h/day [47]. Sleep deprivation has also been associated with overweight/obesity prevalence as well as with higher body fat [47], and increased hunger and appetite [46].

Pattern 3 combined vegetable soup and other “healthy” foods, such as olive oil, starchy foods, and bread, which, in a similar way as pattern 1, resemble the Mediterranean diet pattern [17,48]. Although the consumption of vegetable soup is very important as a mean to increase vegetable intake [49], to decrease the energy density of the diet [50] and to prevent obesity [51,52] no association was found between overweight/obesity and pattern 4 scores. Milk intake may be inversely related to body weight and body fat mass [53], but no association was found between overweight/obesity and food patterns that included milk and milk-based (patterns 6 and 8).

To our knowledge, this is the first report addressing the associations between dietary patterns, sleeping, physical activity/inactivity, parental education and weight, adjusting for confounders (age and energy intake). However, the limitations should be mentioned. First, we were unable to draw cause-effect conclusions and to make observations over the time because of the cross-sectional nature of our data. However, the appropriate analysis of these data can be a valuable initial step for the study of the associations between food intake, lifestyle and obesity. Nonetheless, longitudinal studies are clearly needed to investigate these associations in children. Unfortunately, we do not know if there are children belonging to the same household; it is impossible to account for the effects of correlation between children from the same household.

In addition, the behavioral data, namely the FFQ, relied upon self reported data from parents. Previous studies using and evaluating this methodology suggest that participants may underestimate the amount of fat and protein intakes and significantly overestimate carbohydrate intake [54]. However, although the FFQ may overestimate total intake, it is a good instrument for ranking intakes [55] as intended in our study. In addition, although the FFQ used was validated for Portuguese adults, no validation of this method was accomplished when parents/guardians respond to record their children’s diet, neither were the portion sizes adapted for children. This approach depends on the parents/guardians memory and awareness on what their children eat, but is expected to provide accurate information on the usual average intake [56] in order to rank intakes. No reliability data and validity references were shown for some variables including sociodemographic, sleeping and physical activity variables. In relation to physical activity, we considered several categories to assess the frequency of regular sports activity, and subsequently grouped the categories for the analysis into three categories: less than 2 times/week; 2−3 times/week; 4 or more times/week. Although no reliability and validity measures are available for the questions and categories we used, AHA recommends that adults and children should be active at least four days of the week, and to incorporate increased physical activity into after-school activities [57]. It has also been suggested that studies evaluating physical activity in children may neglect some forms of sedentary behavior [58], and methods for assessing TV viewing have not kept pace with the research interest in the topic [59]. In the present study, a self-report measure to assess TV/video watching was used, as in a previous Portuguese study [3], although research regarding its validity and reliability is lacking.

On the other hand, the present study has important strengths that should be acknowledged. The associations between lifestyle behaviors and diet were assessed examining dietary patterns rather than specific nutrients or foods. Despite the difficulty to map accurately the detailed causes of inadequate nutritional intake in children, given the complexity of dietary behaviors, the current research focusing on clustering whole diet may help to achieve a better understanding of behaviors suspected of having important health outcomes. The strong predictive coefficients identified in the present study for TV viewing, maternal education, sports activities, and sleep duration, create a compelling argument for the important role of these variables in children’s well-being and healthy dietary intake. It is well recognized that changes in dietary behavior may be brought about, not by direct modification of food habits, but by alteration or manipulation of the education and culture [60].

## 5. Conclusion

The present study identified eight major dietary patterns (vegetables, pulses, fruit, olive oil; fish, meat, processed meats, eggs, and starchy foods; vegetable soup, olive oil, butter, starchy foods, and bread; fast-food, SSB, and pastry; olive oil, butter, and margarine; yoghurt, cheese, and ice cream; crackers/cookies, and pastry; milk, milk pudding, and ready to eat cereals). TV viewing (>2 h/day) and male gender were significant positive predictors of pattern 4 (fast-food, SSB, and pastry), while higher level maternal education and longer sleeping duration were positively associated with dietary patterns that included vegetables, pulses, fruit, olive oil, and vegetable soup, and negatively associated with fast-food, SSS and pastry intake. Obesity was negatively associated with the pastry, crackers/cookies food pattern, and positively associated with yogurt, cheese, and ice cream intake. These findings highlighted the importance that maternal education and children sleeping duration may have on healthy eating.

## Figures and Tables

**Table 1.** Nutrient and food intake by gender.

	Girls (n = 991)	Boys (n = 985)	p
Energy (kcal/day)	2177 (593)	2327 (647)	< 0.001
Total fat (% TEI)*	32.3 (5.0)	31.9 (4.5)	0.074
SFA (% TEI)*	10.7 (2.1)	10.6 (1.9)	0.155
MUFA (% TEI)*	13.7 (2.8)	13.5 (2.5)	0.138
PUFA (% TEI)*	5.3 (1.2)	5.2 (1.4)	0.343
Protein (% TEI)*	18.5 (3.5)	18.1 (2.9)	0.025
Total CHO (% TEI)*	51.0 (7.1)	51.7 (6.2)	0.017
Sugars^a^ (% TEI) ^*^	23.3 (6.1)	23.3 (5.7)	0.718
Fibre (g/day)	24.1 (9.9)	25.7 (11.1)	0.001
Vegetables (g/day)	103 (152)	91 (147)	0.024
Vegetable soup (g/day)	295 (358)	295 (358)	0.336
Pulses (g/day)	26 (36)	26 (49)	0.627
Fruit (g/day)	250 (211)	258 (221)	0.228
Yoghurt (g/day)	125 (71)	125 (71)	0.095
Cheese (g/day)	13 (13)	4 (21)	0.586
Milk pudding (g/day)	8 (18)	8 (18)	0.466
Ice cream (g/day)	5 (11)	5 (11)	0.508
Milk (g/day)	610 (366)	610 (366)	0.205
Eggs (g/day)	7 (15)	7 (15)	0.846
Fish (g/day)	58 (51)	56 (50)	0.628
Meat (g/day)	91 (62)	96 (61)	0.039
Processed meats (g/day)	16 (19)	17 (21)	0.027
Bread (g/day)	47 (65)	52 (63)	0.008
Starchy foods (g/day)	163 (81)	169 (96)	0.058
Cereals (g/day)	17 (24)	17 (24)	0.894
Crackers/cookies (g/day)	8 (7)	8 (13)	0.215
Pastry (g/day)	32 (34)	33 (36)	0.057
Fast-food (g/day)	31 (24)	31 (26)	0.064
SSB (g/day)	106 (188)	132 (214)	0.001
Olive oil (g/day)	6 (9)	6 (9)	0.613
Vegetable oils (g/day)	0 (2)	0 (2)	0.362
Margarine (g/day)	0 (0.3)	0 (0.3)	0.798
Butter (g/day)	2 (4)	2 (4)	0.432

Abbreviations: CHO, carbohydrate; MUFA, monounsaturated fatty acid; PUFA, polyunsaturated fatty acid, SFA, saturated fatty acid; SSB – sugar sweetened beverages.

a Sugars refer to all monosaccharides and disaccharides added to foods by the manufacturer, cook or consumer, plus sugar naturally present in honey, syrups, and fruit juices.

* Expressed in % of the total energy intake (TEI).

Data presented as mean (SD), except for food groups expressed as median (interquartile range).

**Table 2.** Characteristics of the children and their parents.

	Girls (n = 991)	Boys (n = 985)	p
**Parents**			
Education			
Father			
≤ 9 years	31.4%	32.9%	
10–12 years	25.5%	21.6%	
> 12 years	43.1%	45.5%	0.138
Mother			
≤ 9 years	29.9%	29.0%	
10–12 years	22.8%	20.8%	
> 12 years	47.3%	50.2%	0.392

**Children**			
Television/video viewing	88.9%	89.7%	
< 2 h/day	11.1%	10.3%	0.543
≥ 2 h			
Sports activities (times/week)			
< 2/week	58.8%	46.8%	
2–3/week	33.0%	39.5%	
4–6/week	6.0%	10.3%	
Every day	2.3%	3.4%	< 0.001
Sleeping (hours/day)			
≤ 8 h/day	21.5%	24.2%	
9 h/day	49.9%	50.1%	
≥ 10 h/day	28.6%	25.7%	0.217
Under weight	2.5%	2.7%	
Normal weight	58.7%	58.6%	
Overweight	27.5%	24.6%	
Obese	11.3%	14.0%	0.488

**Table 3.** Component loadings on food groups.

	Patterns							
	1	2	3	4	5	6	7	8
Variance (%)	9.9	7.1	5.9	5.8	5.3	5.0	4.7	4.3
Vegetables	**0.80**	0.04	0.02	−0.08	0.16	0.01	−0.02	0.09
Vegetable soup	0.22	0.10	**0.47**	−0.15	−0.17	0.01	0.01	0.01
Pulses	**0.71**	0.13	−0.01	0.03	0.01	−0.10	−0.02	0.04
Fruit	**0.56**	−0.02	0.12	0.17	−0.13	0.28	0.05	−0.20
Yoghurt	0.02	−0.06	0.10	−0.08	−0.03	**0.65**	0.18	0.03
Cheese	0.05	0.08	0.24	−0.04	−0.06	**0.40**	−0.09	0.02
Milk pudding	0.01	0.09	−0.27	0.19	0.21	0.24	−0.03	**0.52**
Ice cream	−0.05	0.06	−0.24	0.08	0.04	**0.63**	0.01	0.14
Milk	−0.05	0.10	0.28	−0.23	−0.06	0.20	−0.07	**0.61**
Eggs	−0.05	**0.32**	0.18	0.14	0.15	0.26	−0.18	−0.08
Fish	0.19	**0.56**	0.11	−0.14	−0.05	0.22	0.07	−0.14
Meat	0.01	**0.77**	0.10	0.01	−0.05	−0.02	0.04	0.07
Processed meats	0.05	**0.61**	−0.24	0.19	0.10	−0.07	−0.01	0.28
Bread	0.01	0.06	**0.62**	0.04	0.07	0.05	0.10	0.15
Starchy foods	0.08	0.39	**0.31**	0.13	0.10	−0.02	0.22	−0.08
Cereals (ready-to-eat)	0.11	−0.13	0.22	0.12	−0.17	−0.15	0.29	**0.55**
Crackers/cookies	0.01	0.03	0.06	−0.13	0.06	0.06	**0.77**	0.04
Pastry	−0.06	0.13	−0.02	**0.39**	0.11	0.04	**0.62**	0.01
Fast-food	0.04	0.19	0.04	**0.61**	−0.11	−0.10	−0.02	0.08
SSB	0.01	−0.08	0.04	**0.73**	0.01	0.07	0.06	−0.04
Olive oil	**0.32**	−0.03	**0.32**	−0.10	**0.41**	−0.02	−0.12	0.08
Vegetable oils	0.04	−0.04	0.01	0.05	**0.69**	0.01	0.02	0.02
Margarine	−0.02	0.09	0.03	−0.09	**0.66**	−0.04	0.14	−0.06
Butter	−0.09	0.01	**0.56**	0.27	0.23	0.07	−0.04	−0.03

Notes: SSB—sugar sweetened beverages; factor loadings above 0.30 are in bold numbers.

**Table 4.** Generalized linear models showing relationships between scores of food patterns and gender, physical activity, obesity and parental education.

	β coefficient	Standard error	95% CI	P-value
**Pattern 1 (vegetables, pulses, fruit, olive oil)**				
Sleeping				
≥ 10 h/day	0.099	0.045	0.012, 0.186	0.026
Maternal education				
>12 years	0.107	0.053	0.002, 0.211	0.045
**Pattern 2 (fish, meat, processed meats, eggs, and starchy foods)**				
Boys	0.037	0.018	0.001, 0.073	0.043
TV viewing				
≥ 2 h/day	0.065	0.031	0.004, 0.126	0.037
Sleeping				
≥ 10 h/day	0.083	0.026	0.032, 0.134	0.002
Sports activities				
2–3 times/week	0.043	0.020	0.004, 0.083	0.032
≥ 4 times/week	0.100	0.031	0.040, 0.160	0.001
Maternal education				
>12 years	0.062	0.030	0.002, 0.121	0.042
**Pattern 3 (vegetable soup, olive oil, butter, starchy foods, and bread)**				
Boys	0.051	0.023	0.006, 0.096	0.025
TV viewing				
≥ 2 h/day	−0.101	0.039	.0.177, −0.025	0.009
Sleeping				
9 h	0.129	0.029	0.072, 0.186	< 0.001
≥ 10 h/day	0.203	0.032	0.139, 0.267	< 0.001
Sports activities				
2–3 times/week	0.087	0.025	0.038, 0.136	0.001
≥ 4 times/week	0.094	0.038	0.019, 0.169	0.014
Maternal education				
>12 years	0.106	0.038	0.031, 0.180	0.005
**Pattern 4 (fast-food, SSB, and pastry)**				
Boys	0.135	0.041	0.055, 0.215	0.001
TV viewing				
≥ 2 h/day	0.249	0.070	0.114, 0.385	< 0.001
Sleeping				
9 h/day	−0.125	0.052	−0.227, −0.024	0.016
≥ 10 h/day	−0.215	0.058	−0.328, −0.101	< 0.001
Maternal education				
>12 years	−0.151	0.067	−0.283, −0.019	0.025
**Pattern 5 (olive oil, butter, and margarine)**				
TV viewing				
≥ 2 h/day	0.202	0.082	0.041, 0.363	0.014
**Pattern 6 (yoghurt, cheese, and ice cream)**				
Obesity	0.145	0.056	0.036, 0.255	0.009
Sports activities				
≥ 4 times/week	0.120	0.060	0.002, 0.237	0.046
**Pattern 7 (crackers/cookies, and pastry)**				
Boys	0.120	0.034	0.053, 0.187	< 0.001
Obesity	−0.123	0.052	−0.226, −0.020	0.019
Sports activities				
≥ 4 times/week	−0.159	0.057	−0.271, −0.048	0.005
**Pattern 8 (milk, milk pudding, and ready to eat cereals)**				
Sleeping				
9 h/day	0.107	0.041	0.026, 0.188	0.009
≥ 10 h/day	0.153	0.046	0.062, 0.243	0.001

Notes: In the generalized linear models, children are nested within schools; independent variables considered children’s gender (omitted/reference category: female), overweight, obesity (omitted/reference category: normal weight), TV viewing (omitted/reference category: up to 2 h/day), sleeping (omitted/reference category: ≤8 h/day), frequency of sports activities (omitted/reference category: <2 times/week), and maternal and paternal education (omitted/reference category: up to 9 years), adjusting for children’s age and energy intake.
